# Supplementary material for: Use of Generalized Weighted Quantile Sum Regressions of Tumor Necrosis Factor Alpha and Kidney Function to Explore Joint Effects of Multiple Metals in Blood
Source: Int J Environ Res Public Health. 2022 Jun 16;19(12):7399. doi: 10.3390/ijerph19127399 (PMC9223707; doi:10.3390/ijerph19127399)
Supplement: Supplementary file 1 [file ijerph-19-07399-s001.zip › ijerph-1725046-supplementary.pdf]

Supplemental Material

Using Generalized Weighted Quantile Sum Regressions of Tumor Necrosis Factor Alpha and Kidney Function through Joint Effects with Multiple Metals Exposure

Contents

Figure S1 Forest plot of single metal effects for TNF-α after adjusting covariates.

Figure S2 Forest plot of single metal effects for TNF-α after adjusting covariates and WBC.

Figure S3 Forest plot of single metal effects for WBC after adjusting covariates.

Figure S4 Forest plot of single metal effects for serum creatinine after adjusting covariates.

Figure S5 Forest plot of single metal effects for eGFR after adjusting covariates.

Figure S6 Forest plot of single metal effects for the ratio of TNF-α and WBC after adjusting covariates.

Figure S7 After adjusting covariate plots for the mean predicted value of the toxic metals mixture index in serum and measures for TNF-α (a), WBC (b), Serum creatinine (c), TNF-α/WBC (d), and eGFR (e).

Figure S8 After adjusting covariate plots for the mean predicted value of the essential metals mixture index in serum and measures for TNF-α (a), WBC (b), Serum creatinine (c), TNF-α/WBC (d), and eGFR (e).

Figure S9 After adjusting covariate plots for the mean predicted value of the seven metals mixture index in serum and measures for TNF-α (a), WBC (b), Serum creatinine (c), TNF-α/WBC (d), and eGFR (e).

Table S1. Associations of single serum metal levels with the TNF-α using regression models.

Table S2. Associations of single serum metal levels with WBC using regression models.

Table S3. Associations of single serum metal levels with serum creatinine using regression models.

Table S4 Associations of single serum metal levels with estimated Glomerular filtration rate (eGFR) using regression models.

Table S5 Associations of single serum metal levels with the ratio of TNF-α and WBC using regression models.

Table S6 Results of toxic metals mixture by WQS regression analyses with covariates.

Table S7 Results of essential metals mixture by WQS regression analyses with covariates.

Table S8 Results of all metals mixture by WQS regression analyses with covariates.

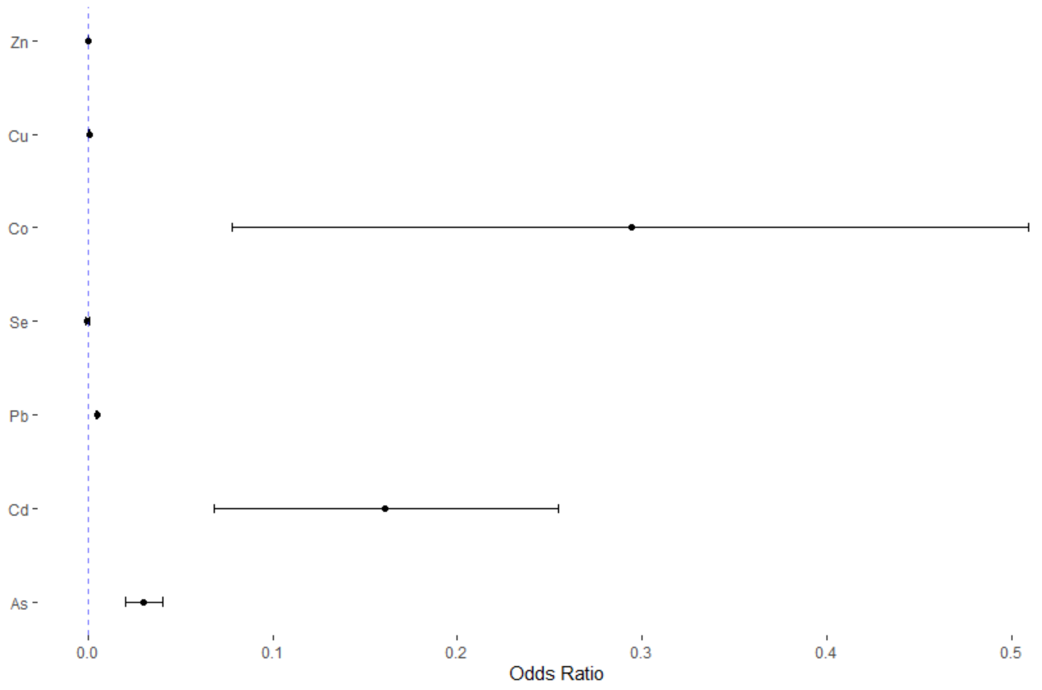

Figure S1. Forest plot of single metal effects for TNF-α after adjusting covariates.

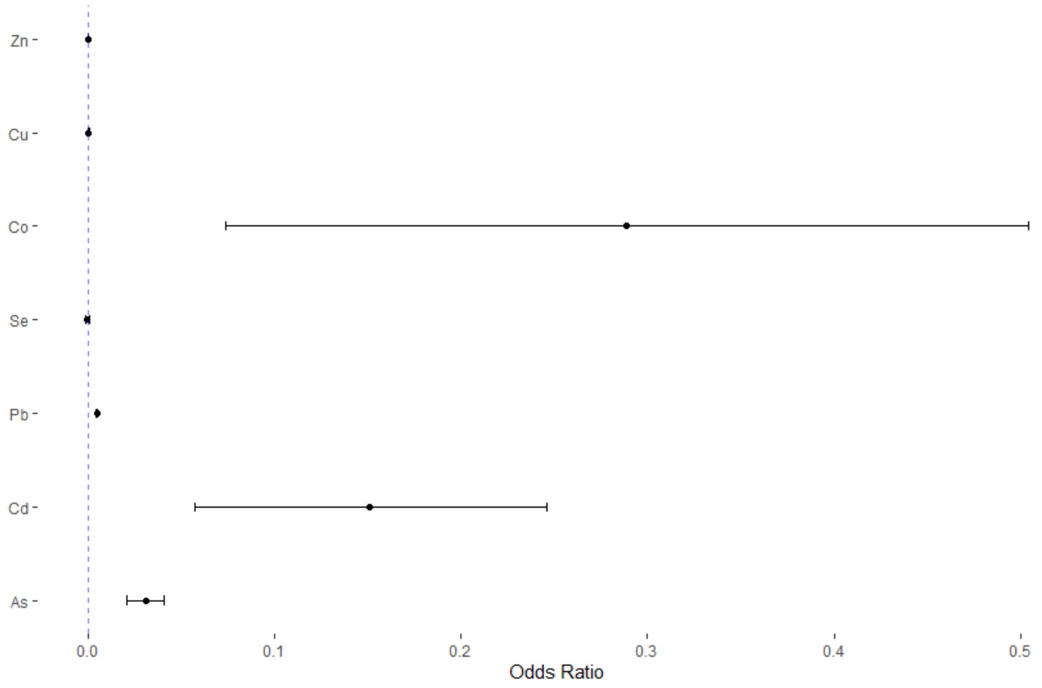

Figure S2. Forest plot of single metal effects for TNF-α after adjusting covariates and WBC.

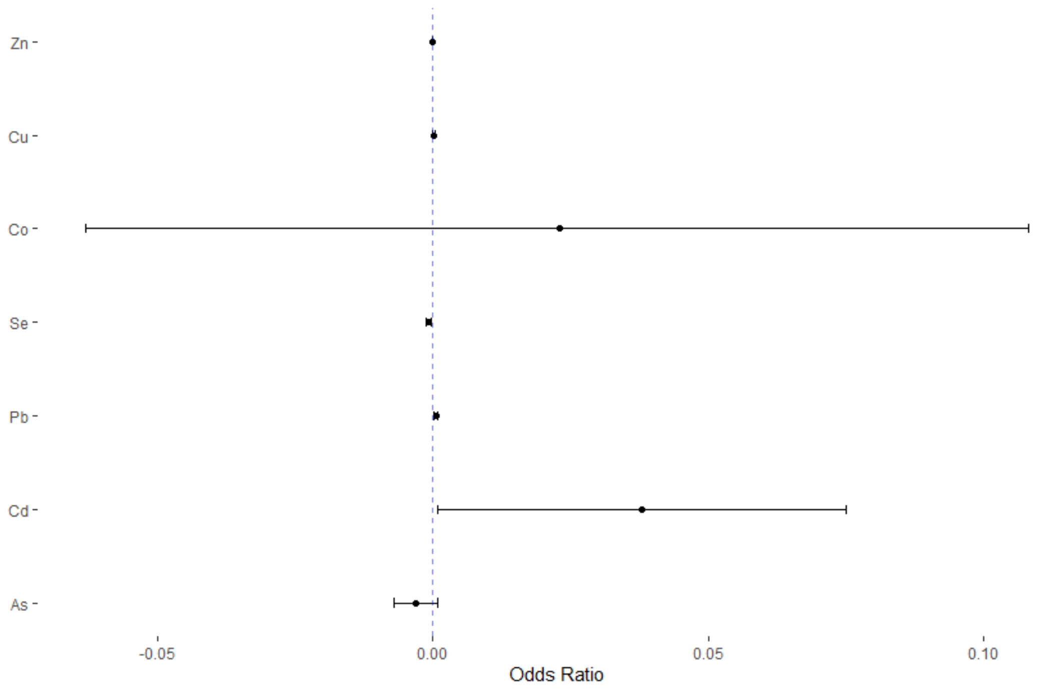

Figure S3. Forest plot of single metal effects for WBC after adjusting covariates.

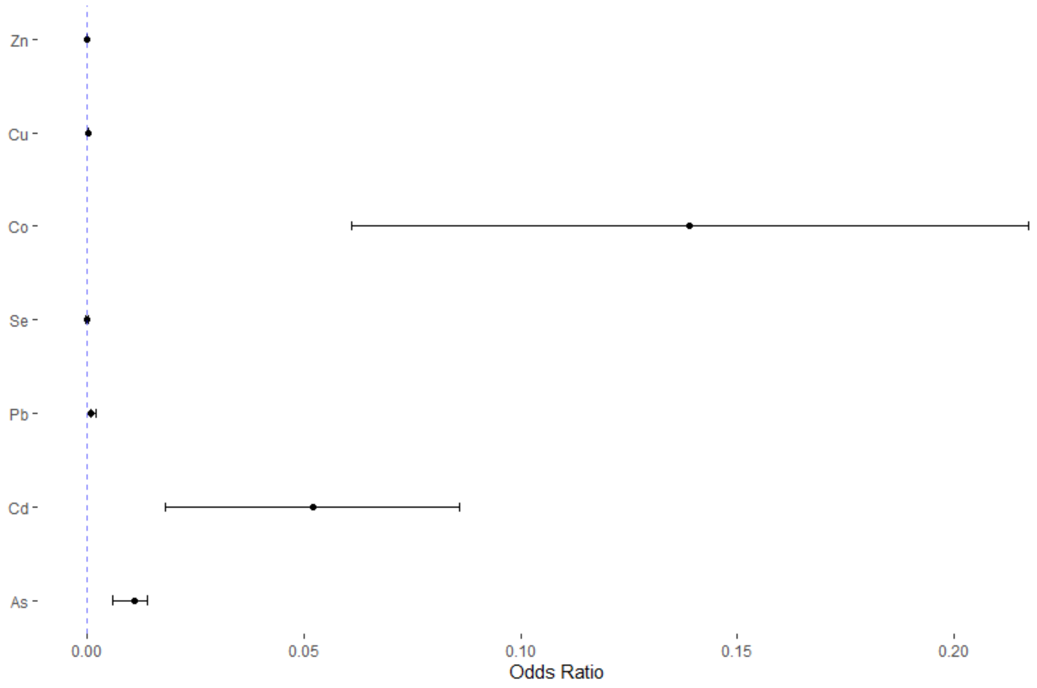

Figure S4. Forest plot of single metal effects for serum creatinine after adjusting covariates.

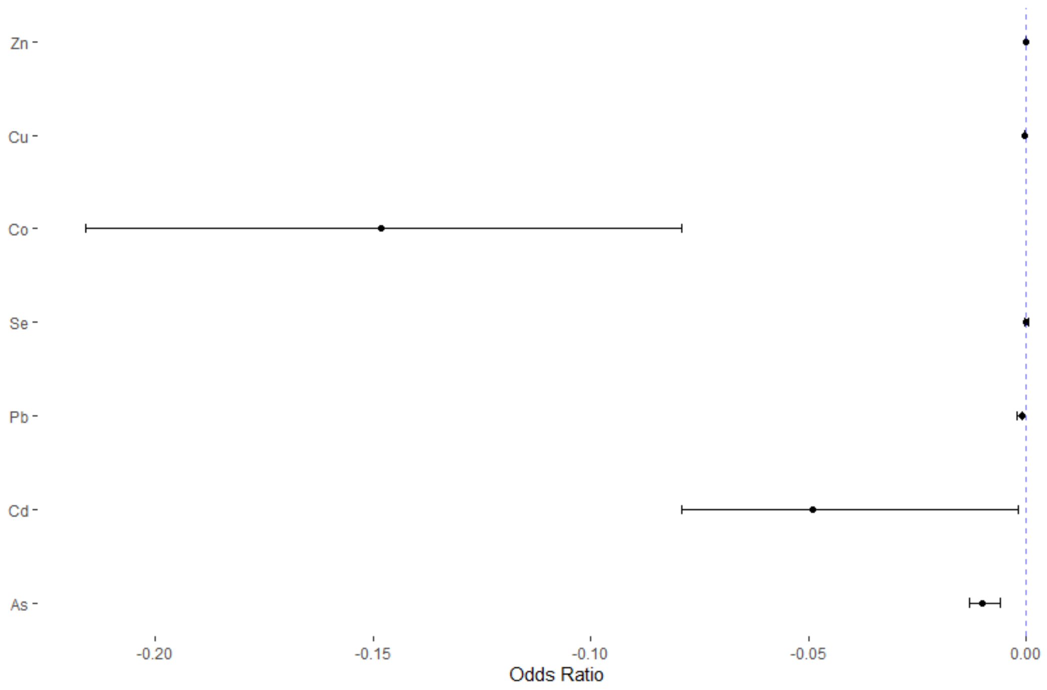

Figure S5. Forest plot of single metal effects for eGFR after adjusting covariates.

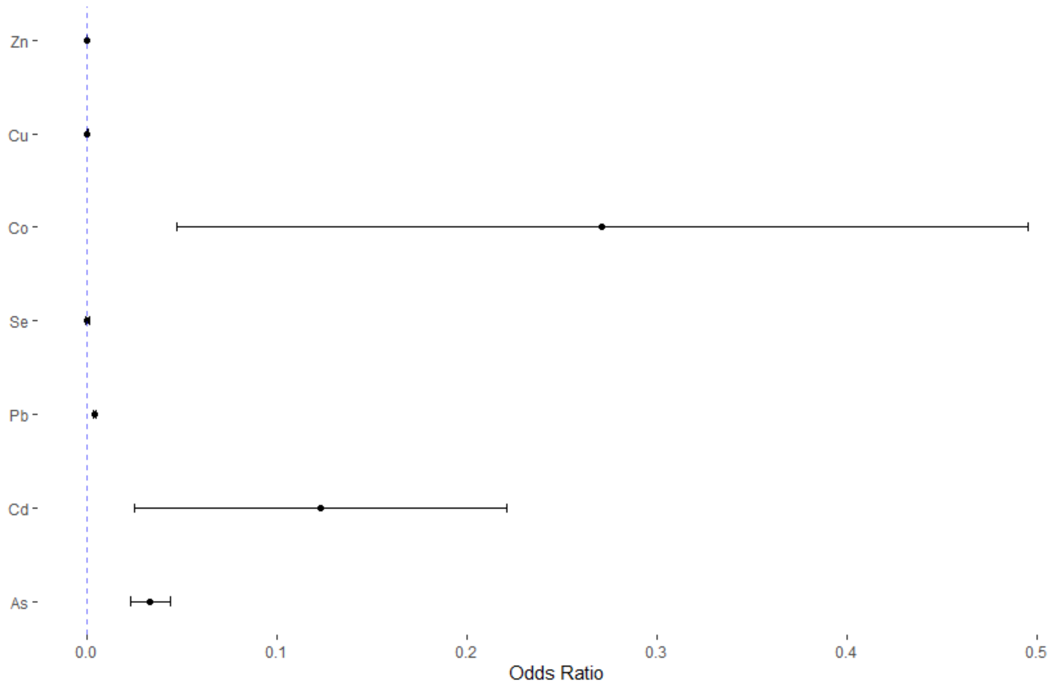

Figure S6. Forest plot of single metal effects for the ratio of TNF-α and WBC after adjusting covariates.

Table S1. Associations of single serum metal levels with the TNF-α using regression models.

Metals	Model 1			Model 2[a]			Model 3 [b]		
	Regression Coefficient	(95% CI)	*P* value	Regression Coefficient	β (95% CI)	*P* value	Regression Coefficient	β (95% CI)	*P* value
Arsenic (As)	0.032	0.022, 0.042	<0.001	0.030	0.020, 0.040	<0.001	0.031	0.021, 0.041	<0.001
Cadmium (Cd)	0.160	0.076, 0.236	<0.001	0.161	0.068, 0.255	<0.001	0.151	0.057, 0.246	0.002
Lead (Pb)	0.005	0.004, 0.005	<0.001	0.005	0.004, 0.005	<0.001	0.005	0.004, 0.005	<0.001
Selenium (Se)	-0.00008	-0.0012, 0.0010	0.891	-0.0004	-0.0016, 0.0007	0.476	-0.0002	-0.0014, 0.0009	0.697
Cobalt (Co)	0.300	0.088, 0.511	0.006	0.294	0.078, 0.509	0.007	0.289	0.074, 0.504	0.009
Copper (Cu)	0.0005	0.0002, 0.0008	0.003	0.0005	0.0002, 0.0008	0.003	0.0004	0.0001, 0.0008	0.008
Zinc (Zn)	0.00009	0.00006, 0.00011	<0.001	0.00009	0.00006, 0.00011	<0.001	0.00008	0.00006, 0.00011	<0.001

[a] After adjusting for age, gender, BMI, smoking status and drinking status.

[b] After adjusting for age, gender, BMI, WBC, smoking status and drinking status.

Table S2. Associations of single serum metal levels with WBC using regression models.

Metals	Model 1			Model 2[a]		
	Regression Coefficient	(95% CI)	*P* value	Regression Coefficient	β (95% CI)	*P* value
Arsenic (As)	-0.003	-0.007, 0.001	0.191	-0.003	-0.007, 0.001	0.130
Cadmium (Cd)	0.063	0.029, 0.096	<0.001	0.038	0.001, 0.075	0.043
Lead (Pb)	0.0007	0.0005, 0.0010	<0.001	0.0006	0.0003, 0.0009	0.001
Selenium (Se)	-0.0005	-0.00096, -0.00004	0.032	-0.0008	-0.0012, -0.0003	0.001
Cobalt (Co)	-0.043	-0.132, 0.046	0.345	0.023	-0.063, 0.108	0.602
Copper (Cu)	0.0002	0.00007, 0.00033	0.003	0.0002	0.0001, 0.0004	<0.001
Zinc (Zn)	0.00002	0.000008, 0.00003	0.001	0.00002	0.000005, 0.000027	0.004

[a] After adjusting for age, gender, BMI, smoking status and drinking status.

Table S3. Associations of single serum metal levels with serum creatinine using regression models.

Metals	Model 1			Model 2[a]		
	Regression Coefficient	(95% CI)	*P* value	Regression Coefficient	β (95% CI)	*P* value
Arsenic (As)	0.010	0.006, 0.015	<0.001	0.011	0.006, 0.014	<0.001
Cadmium (Cd)	0.058	0.023, 0.093	0.001	0.052	0.018, 0.086	0.003
Lead (Pb)	0.002	0.001, 0.002	<0.001	0.00132	0.00109, 0.00157	<0.001
Selenium (Se)	0.0003	-0.0001, 0.0008	0.156	-0.000001	-0.0004, 0.0004	0.998
Cobalt (Co)	0.030	-0.062, 0.122	0.870	0.139	0.061, 0.217	<0.001
Copper (Cu)	0.0002	0.00005, 0.0003	0.008	0.0003	0.0002, 0.0004	<0.001
Zinc (Zn)	0.00006	0.00005, 0.0007	<0.001	0.00005	0.00004, 0.00006	<0.001

[a] After adjusting for age, gender, BMI, smoking status and drinking status.

Table S4. Associations of single serum metal levels with estimated Glomerular filtration rate (eGFR) using regression models.

Metals	Model 1			Model 2[a]		
	Regression Coefficient	(95% CI)	*P* value	Regression Coefficient	β (95% CI)	*P* value
Arsenic (As)	-0.011	-0.015, -0.008	<0.001	-0.010	-0.013. -0.006	<0.001
Cadmium (Cd)	-0.062	-0.088, -0.035	<0.001	-0.049	-0.079, -0.019	0.002
Lead (Pb)	-0.001	-0.002, -0.001	<0.001	-0.001	-0.002, -0.001	<0.001
Selenium (Se)	-0.0003	-0.0006, 0.00007	0.115	0.0001	-0.0003, 0.0004	0.757
Cobalt (Co)	-0.126	-0.197, -0.056	0.007	-0.148	-0.216, -0.079	<0.001
Copper (Cu)	-0.0003	-0.0004, -0.0002	<0.001	-0.0003	-0.0004, -0.0002	<0.001
Zinc (Zn)	-0.00005	-0.00006, -0.00004	<0.001	-0.00005	-0.00006, -0.00004	<0.001

[a] After adjusting for age, gender, BMI, smoking status and drinking status.

Table S5. Associations of single serum metal levels with the ratio of TNF-α and WBC using regression models.

Metals	Model 1			Model 2[a]		
	Regression Coefficient	(95% CI)	*P* value	Regression Coefficient	β (95% CI)	*P* value
Arsenic (As)	0.035	0.024, 0.046	<0.001	0.033	0.023, 0.044	<0.001
Cadmium (Cd)	0.093	0.008, 0.179	0.033	0.123	0.025, 0.221	0.014
Lead (Pb)	0.004	0.003, 0.004	<0.001	0.0043	0.0036, 0.0049	<0.001
Selenium (Se)	0.0004	-0.000, 0.0016	0.475	0.0003	-0.0009, 0.0015	0.587
Cobalt (Co)	0.342	0.119, 0.565	0.003	0.271	0.047, 0.495	0.018
Copper (Cu)	0.0003	-0.00007, 0.00061	0.119	0.0003	-0.00008, 0.0006	0.140
Zinc (Zn)	0.00007	0.00004, 0.00010	<0.001	0.00007	0.00004, 0.00010	<0.001

[a] After adjusting for age, gender, BMI, smoking status and drinking status.

a

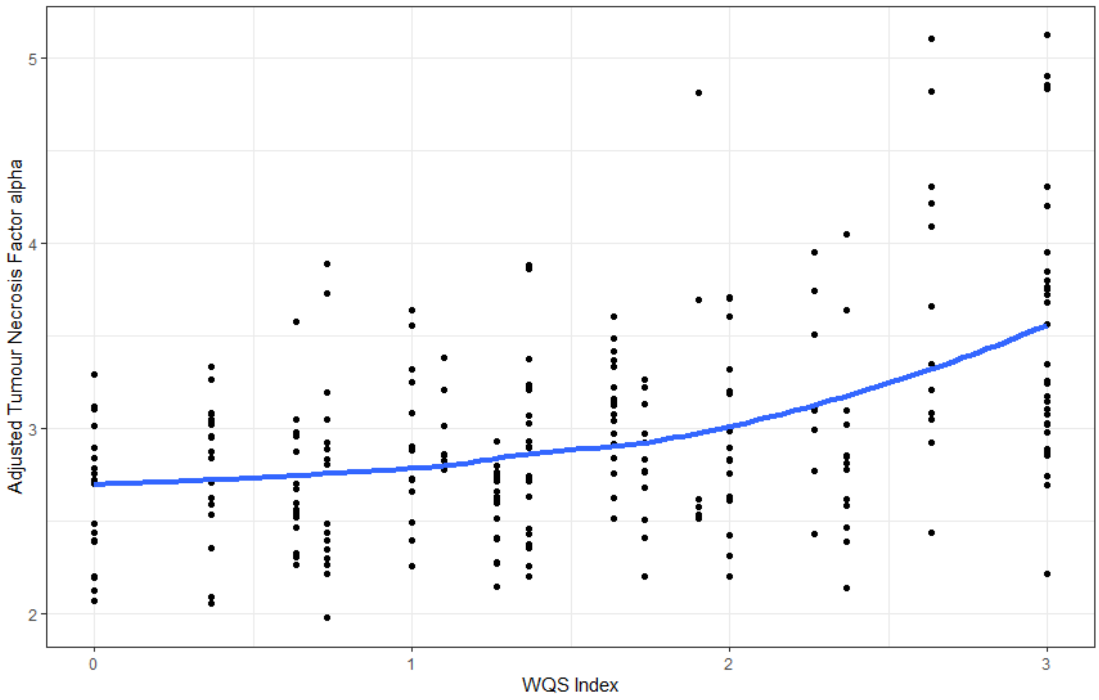

b

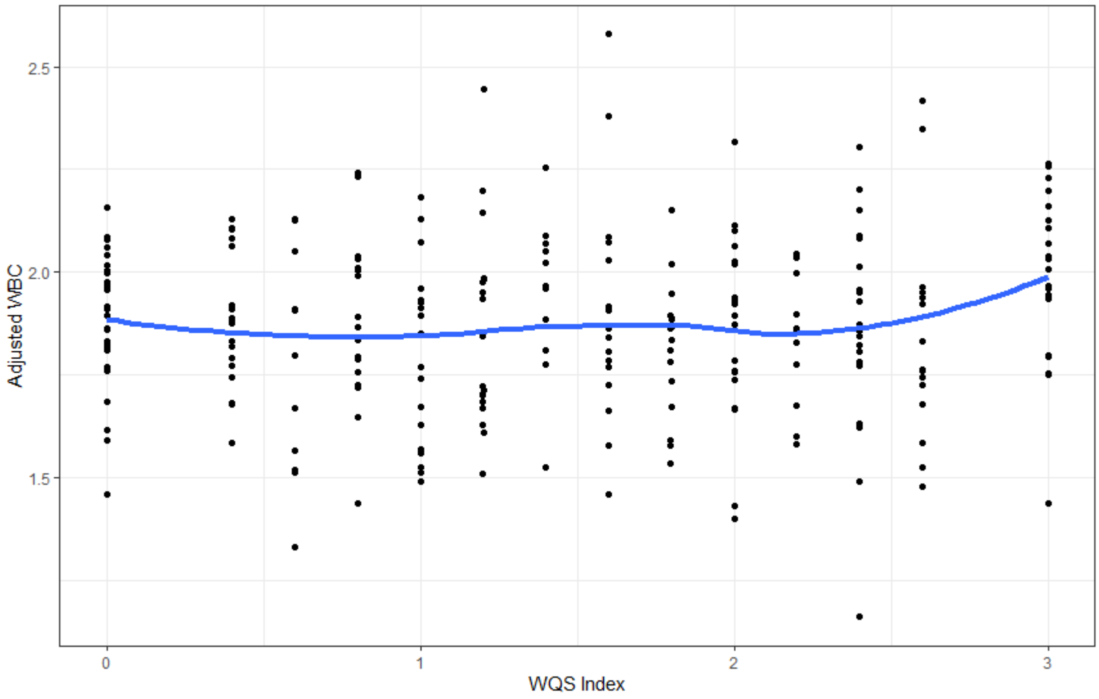
2.5
2.0
1.5
Adjusted WBC
0
1
2
3
WQS Index

c

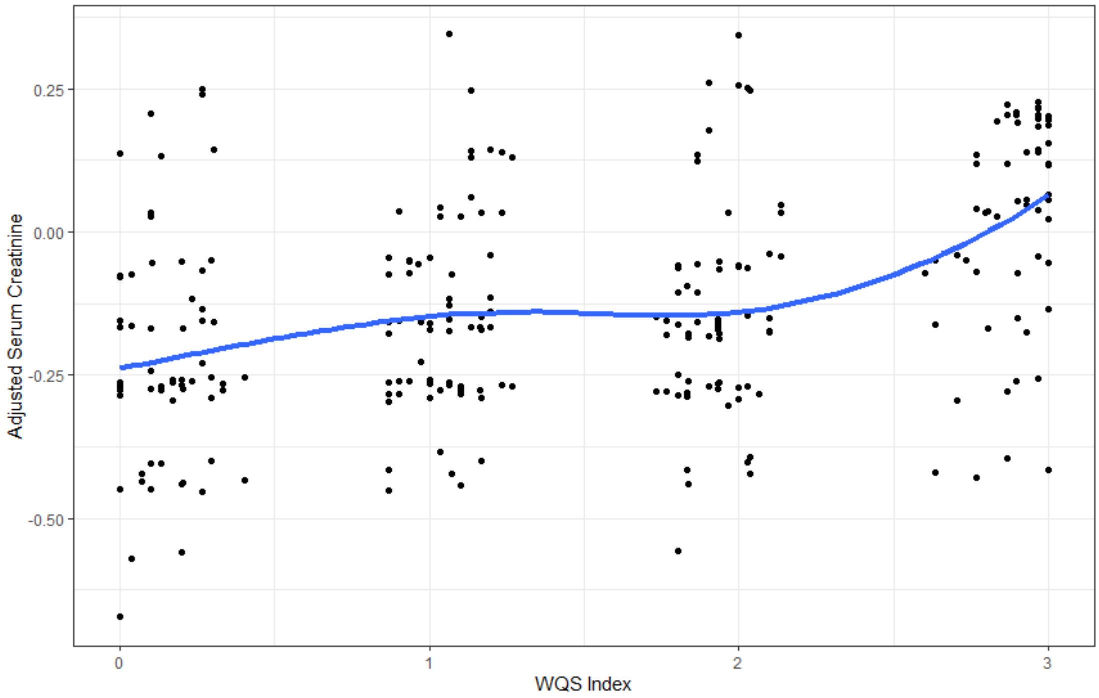
0.25
0.00
-0.25
-0.50
Adjusted Serum Creatinine
0
1
2
3
WQS Index

d

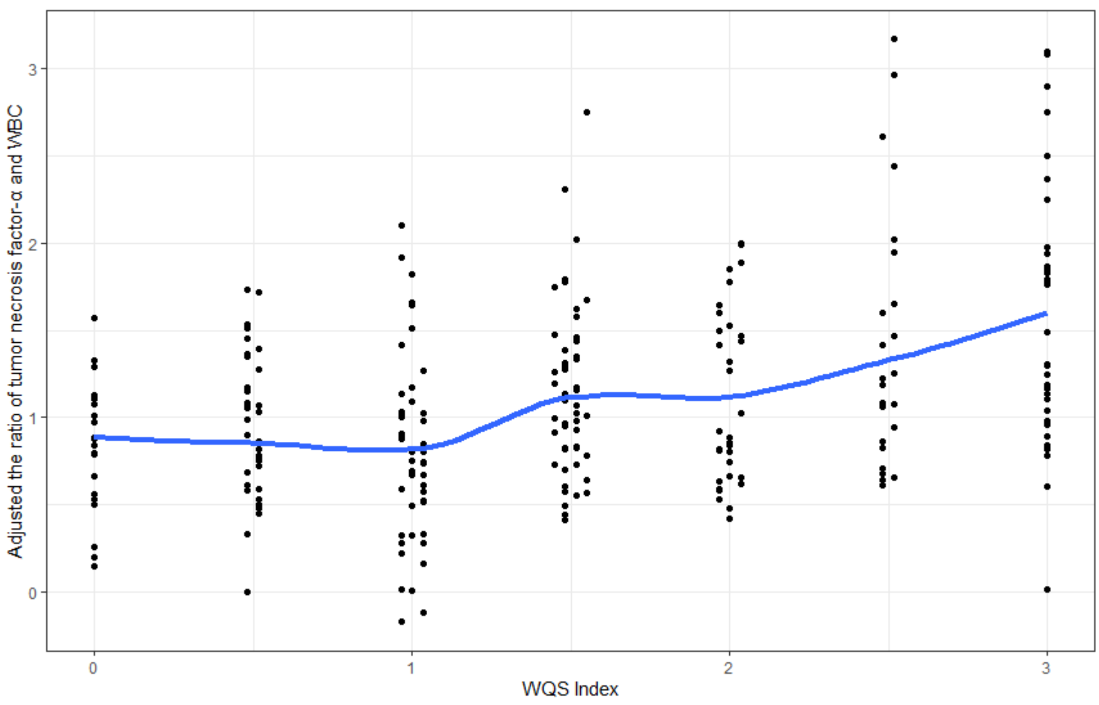

e

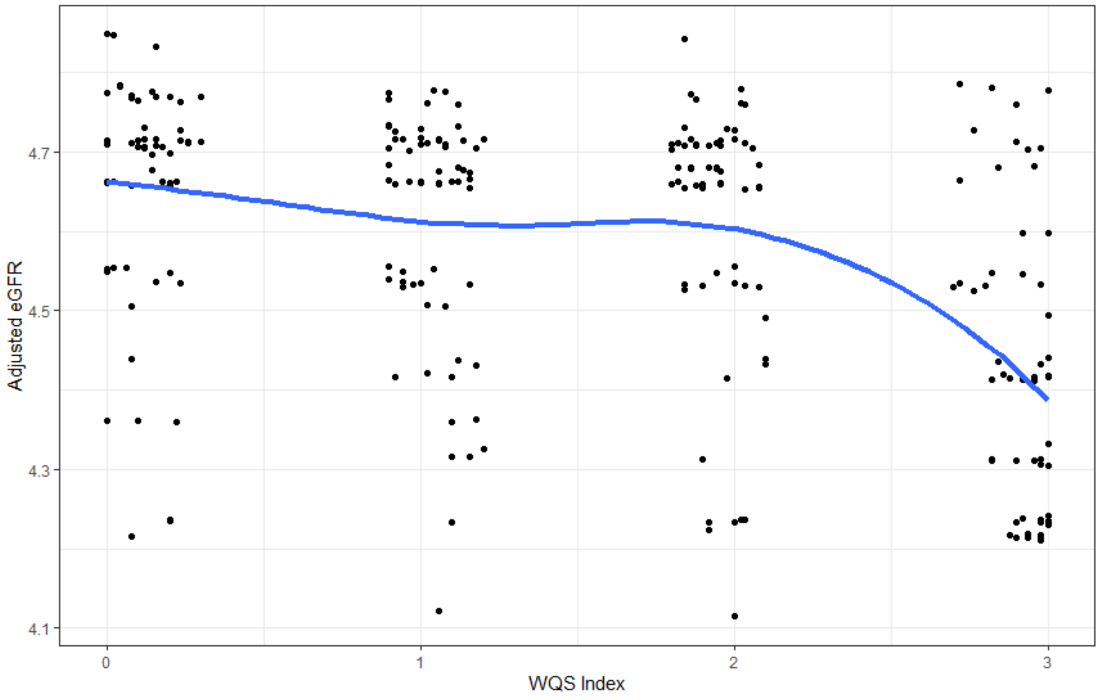

Figure S7. After adjusting covariate plots for the mean predicted value of the toxic metals mixture index in serum and measures for TNF-α (a), WBC (b), Serum creatinine (c), TNF-α/WBC (d), and eGFR (e).

Table S6. Results of toxic metals mixture by WQS regression analyses with covariates.

Outcome	Estimates (95% CI)	p-value
**TNF-α**		
WQS index	0.314 (0.241, 0.387)	<0.001
Gender	-0.008 (-0.188, 0.173)	0.934
Age	0.006 (-0.001, 0.014)	0.106
BMI	-0.023 (-0.043, -0.003)	0.025
Smoking status	0.016 (-0.133, 0.166)	0.833
Drinking status	-0.199(-0.666, 0.268)	0.405
**White blood cells**		
WQS index	0.023 (-0.010, 0.056)	0.168
Gender	0.008 (-0.069, 0.085)	0.835
Age	-0.002 (-0.005, 0.001)	0.244
BMI	0.015 (0.006, 0.023)	0.001
Smoking status	0.090 (0.023, 0.157)	0.009
Drinking status	0.003 (-0.197, 0.202)	0.980
**TNF-α/WBC**		
WQS index	0.279 (0.120, 0.358)	<0.001
Gender	0.011 (-0.182, 0.204)	0.908
Age	0.008 (-0.0004, 0.016)	0.062
BMI	-0.035 (-0.056, -0.013)	0.002
Smoking status	-0.079 (-0.239, 0.082)	0.337
Drinking status	-0.205 (-0.706, 0.296)	0.424
**Serum creatinine**		
WQS index	0.094 (0.070, 0.118)	<0.001
Gender	0.252 (0.187, 0.317)	<0.001
Age	-0.002 (-0.005, 0.0003)	0.085
BMI	-0.002 (-0.009, 0.005)	0.539
Smoking status	-0.001 (-0.054, 0.053)	0.983
Drinking status	0.132 (-0.035, 0.299)	0.123
**eGFR**		
WQS index	-0.087 (-0.108, -0.066)	<0.001
Gender	0.021 (-0.036, 0.079)	0.467
Age	-0.005 (-0.008, -0.003)	<0.001
BMI	0.004 (-0.002, 0.011)	0.176
Smoking status	-0.004 (-0.052, 0.043)	0.864
Drinking status	-0.127 (-0.275, 0.021)	0.093

a

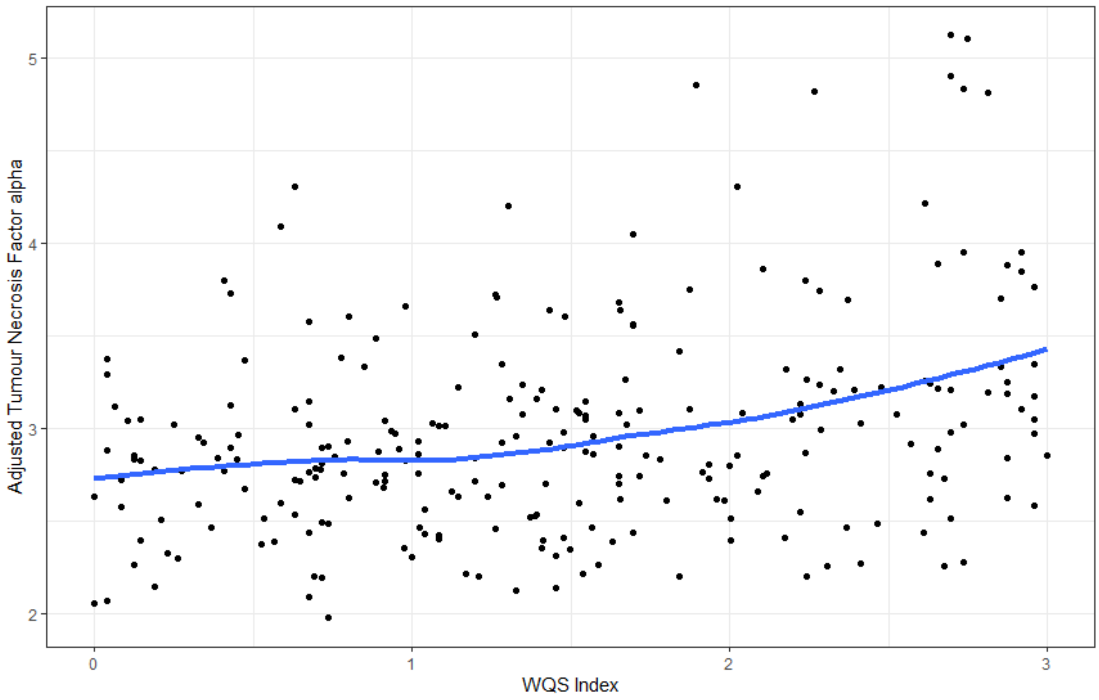

Adjusted Tumour Necrosis Factor alpha
5
4
3
2
0
1
2
3
WQS Index

b

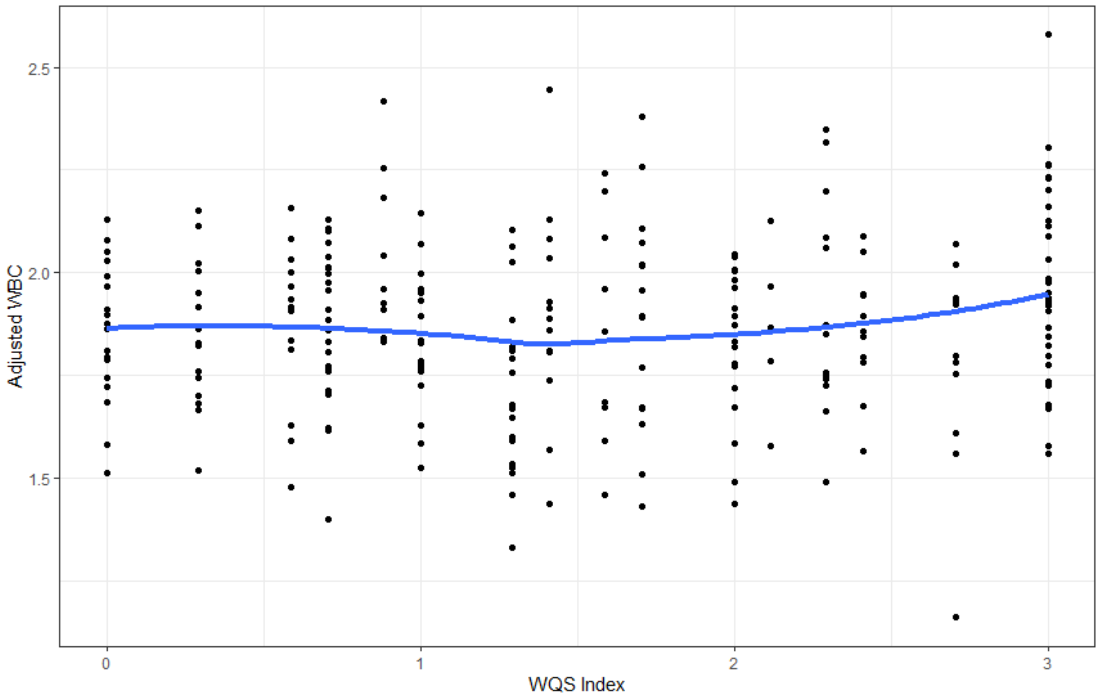

Adjusted WBC
2.5
2.0
1.5
0
1
2
3
WQS Index

c

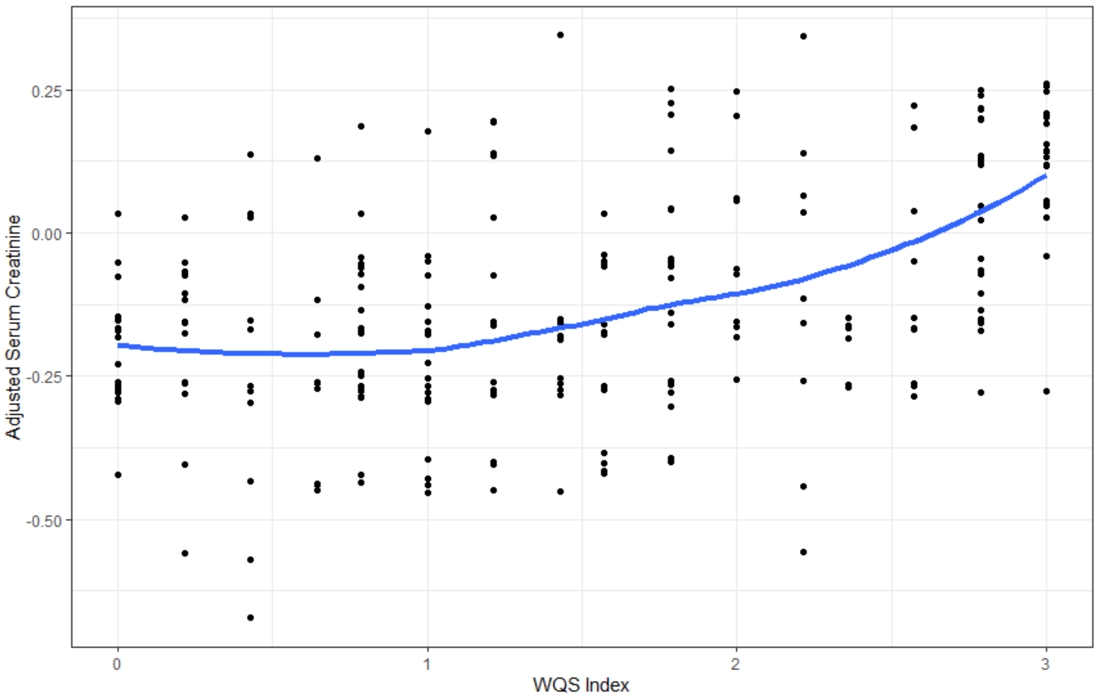
0.25
0.00
-0.25
-0.50
Adjusted Serum Creatinine
0
1
2
3
WQS Index

d

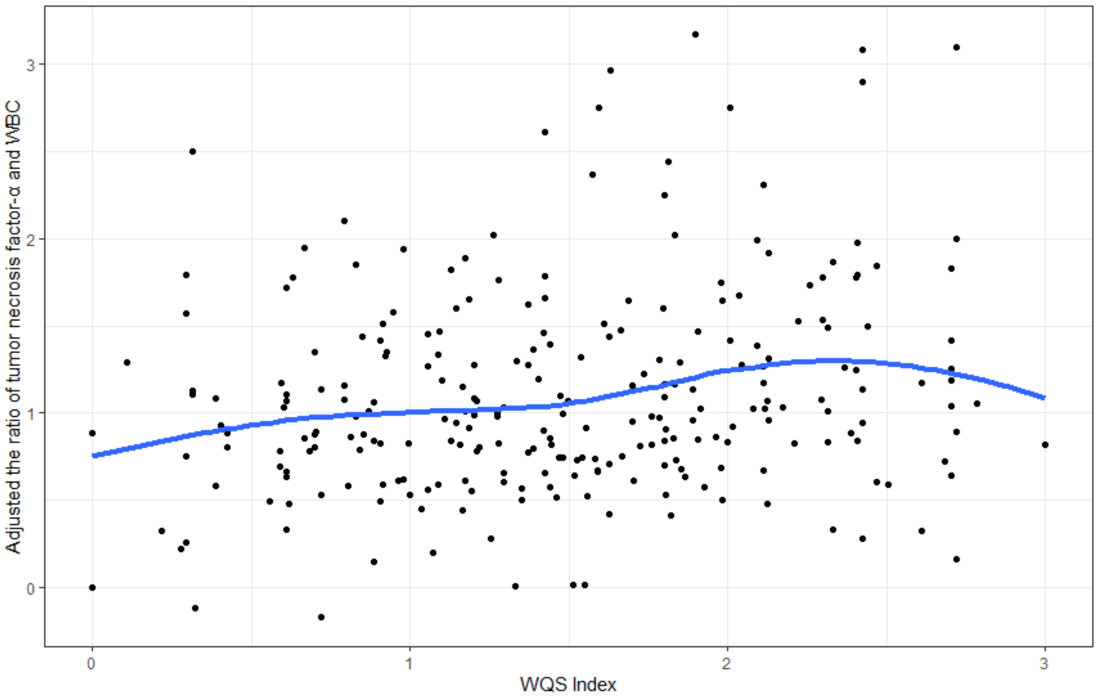
3
2
1
0
Adjusted the ratio of tumor necrosis factor-α and WBC
0
1
2
3
WQS Index

e

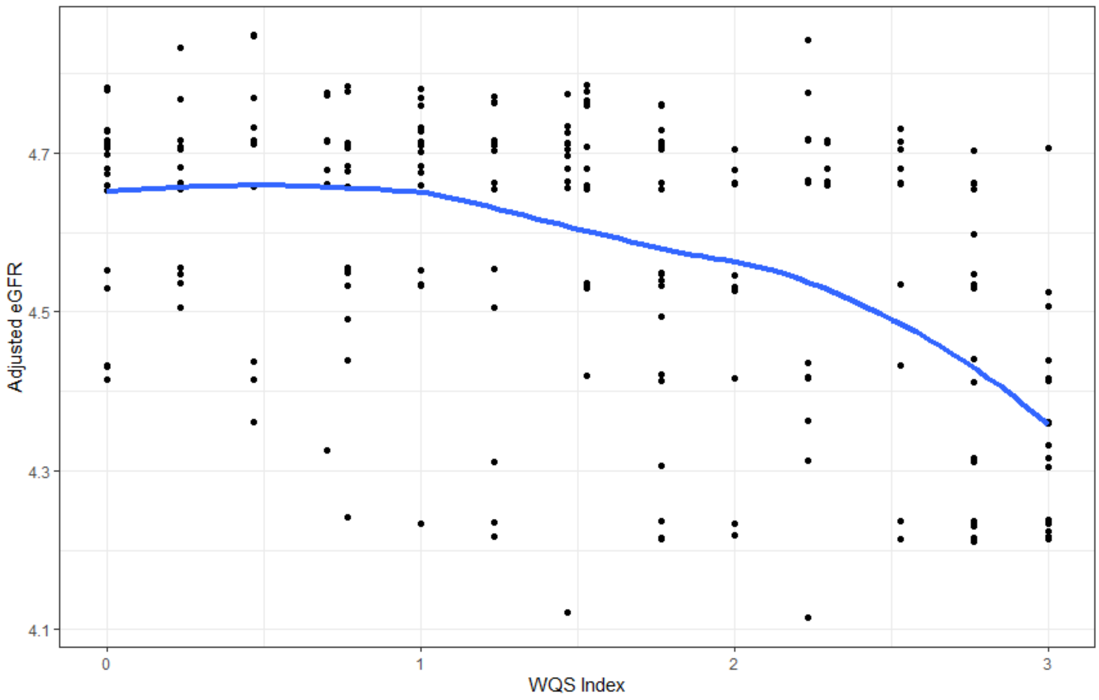

Figure S8. After adjusting covariate plots for the mean predicted value of the essential metals mixture index in serum and measures for TNF-α (a), WBC (b), Serum creatinine (c), TNF-α/WBC (d), and eGFR (e).

Table S7. Results of essential metals mixture by WQS regression analyses with covariates.

Outcome	Estimates (95% CI)	p-value
**TNF-α**		
WQS index	0.217 (0.136, 0.298)	<0.001
Gender	0.103 (-0.089, 0.296)	0.294
Age	0.012 (0.004, 0.021)	0.004
BMI	-0.014 (-0.035, 0.007)	0.204
Smoking status	0.101 (-0.061, 0.263)	0.223
Drinking status	-0.119 (-0.621, 0.383)	0.642
**White blood cells**		
WQS index	0.019 (-0.010, 0.049)	0.201
Gender	0.010 (-0.066, 0.049)	0.794
Age	-0.001 (-0.005, 0.002)	0.393
BMI	0.015 (0.006,0.023)	0.001
Smoking status	0.110 (0.045,0.174)	0.001
Drinking status	0.001 (-0.199, 0.201)	0.992
**TNF-α/WBC**		
WQS index	0.194 (0.084, 0.304)	0.001
Gender	0.114 (-0.090, 0.319)	0.275
Age	0.012 (0.003, 0.021)	0.008
BMI	-0.033 (-0.056, -0.010)	0.004
Smoking status	-0.024 (-0.196, 0.147)	0.782
Drinking status	-0.106 (-0.639,0.427)	0.697
**Serum creatinine**		
WQS index	0.099 (0.075, 0.123)	<0.001
Gender	0.281 (0.217, 0.344)	0.001
Age	-0.001 (-0.004, 0.002)	0.572
BMI	0.003 (-0.004, 0.010)	0.420
Smoking status	0.046 (-0.008, 0.099)	0.096
Drinking status	0.118 (-0.048, 0.284)	0.164
**eGFR**		
WQS index	-0.093 (-0.115, -0.072)	<0.001
Gender	-0.009 (-0.065, 0.048)	0.762
Age	-0.007 (-0.009, -0.004)	<0.001
BMI	-0.001 (-0.007, 0.006)	0.861
Smoking status	-0.046 (-0.094, 0.001)	0.056
Drinking status	-0.114 (-0.261, 0.33)	0.129

a

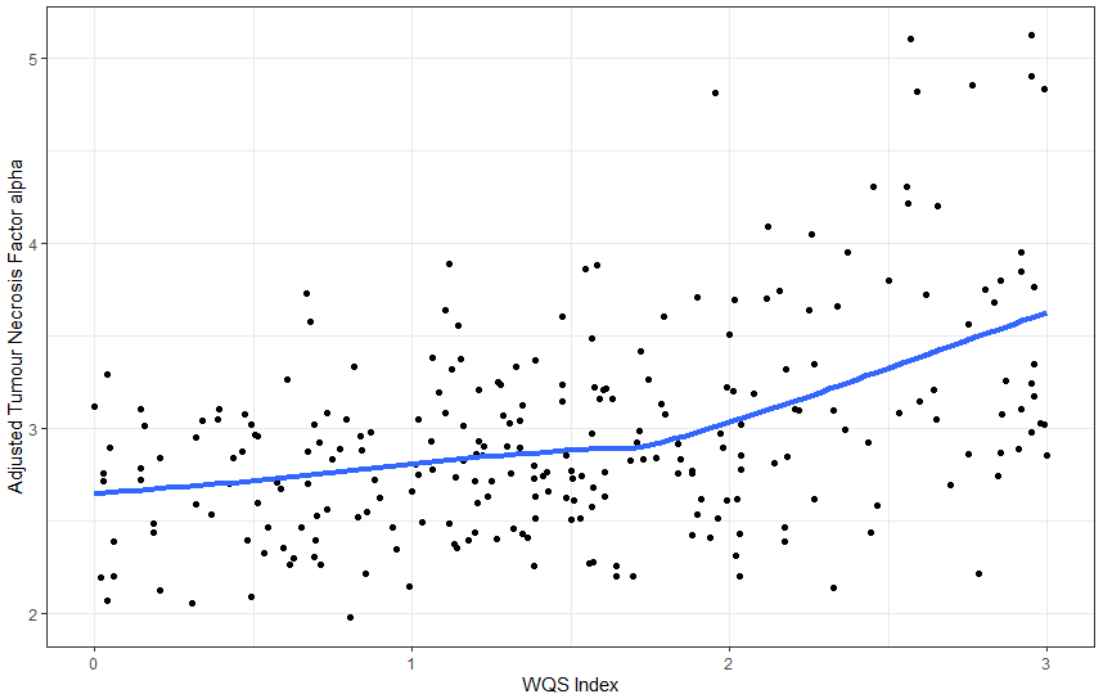

Adjusted Tumour Necrosis Factor alpha
WQS Index
5
4
3
2
0
1
2
3

b

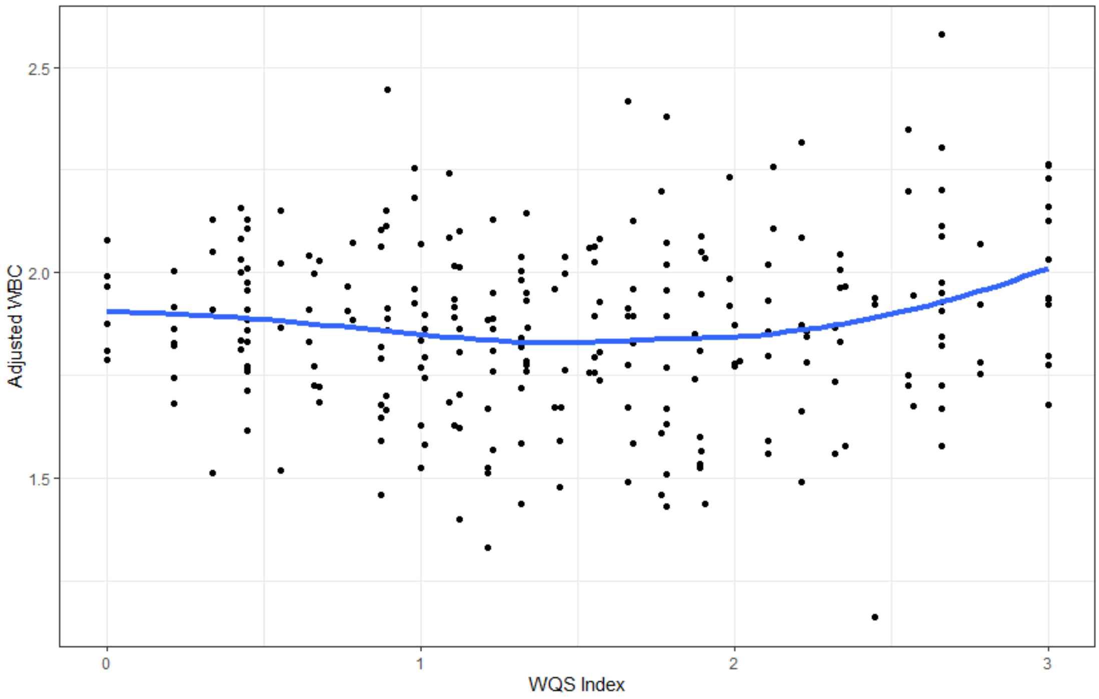

Adjusted WBC
WQS Index
2.5
2.0
1.5
0
1
2
3

c

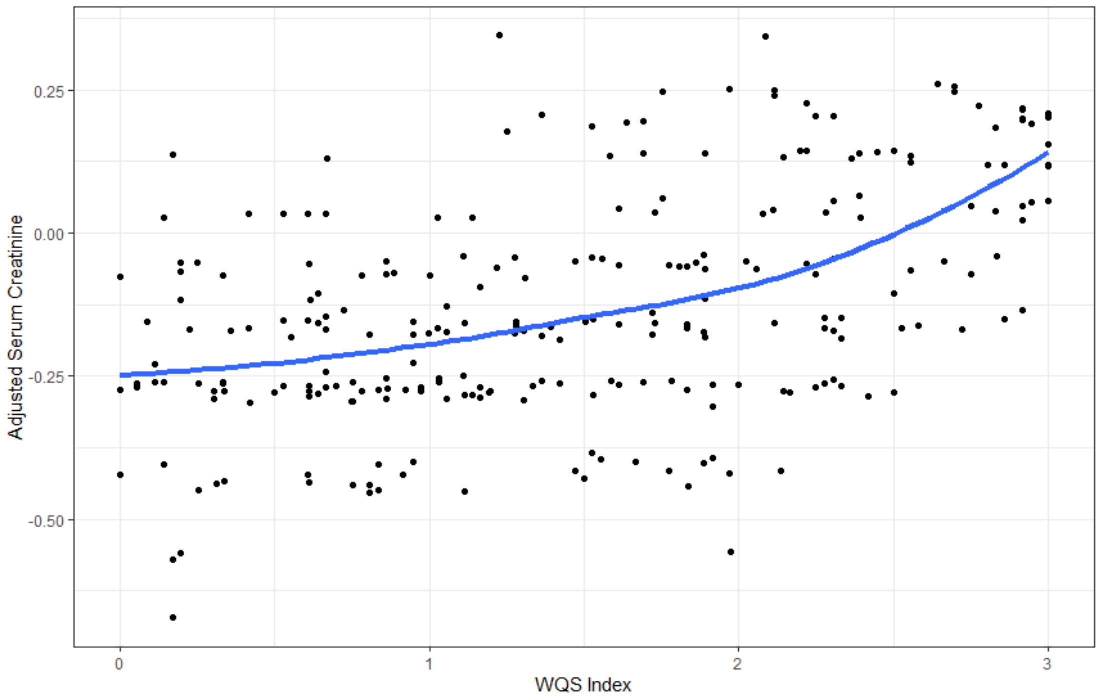

Adjusted Serum Creatinine
0.25
0.00
-0.25
-0.50
0
1
2
3
WQS Index

d

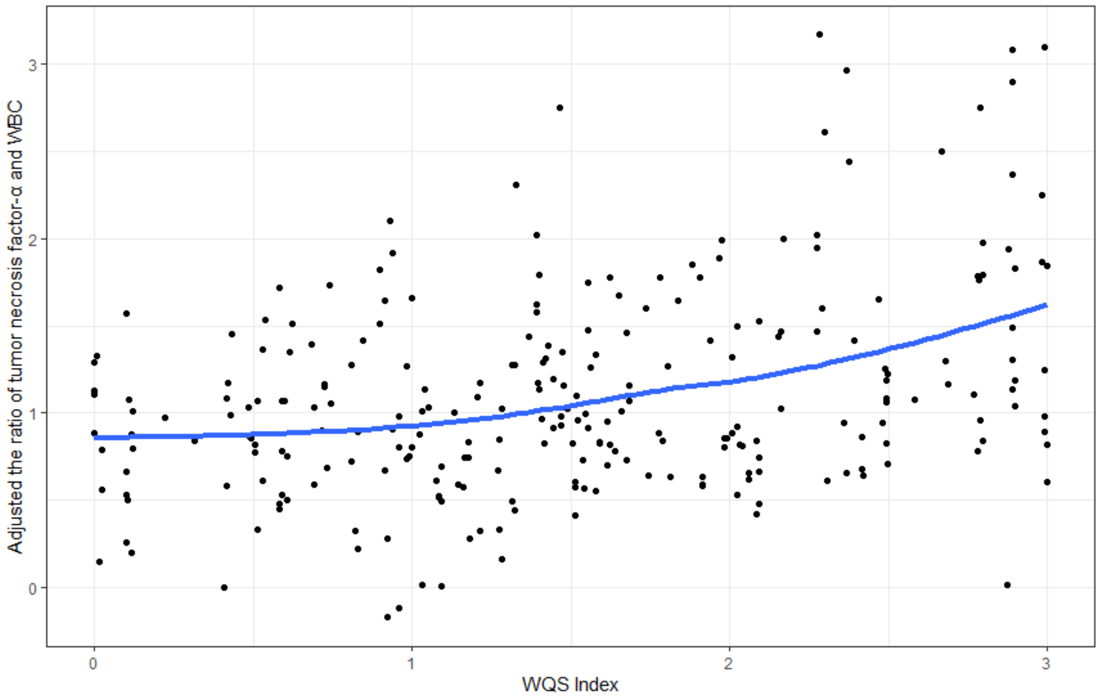

Adjusted the ratio of tumor necrosis factor-α and WBC
3
2
1
0
0
1
2
3
WQS Index

e

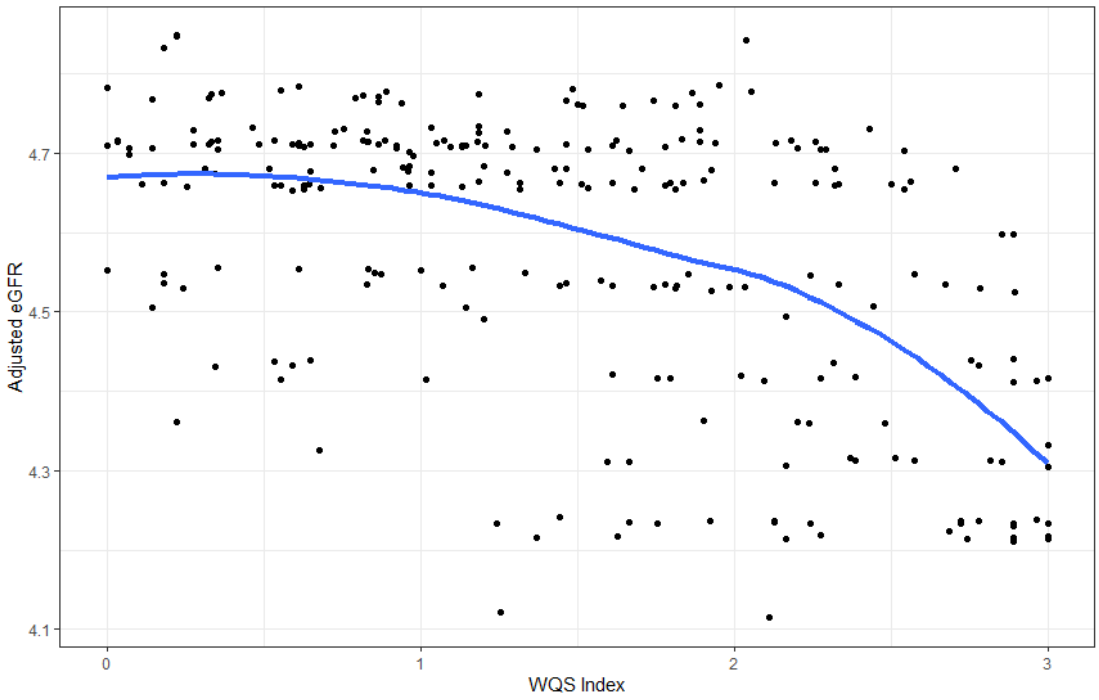

Figure S9. After adjusting covariate plots for the mean predicted value of the seven metals mixture index in serum and measures for TNF-α (a), WBC (b), Serum creatinine (c), TNF-α/WBC (d), and eGFR (e).

Table S8. Results of all metals mixture by WQS regression analyses with covariates.

Outcome	Estimates (95% CI)	p-value
**TNF-α**		
WQS index	0.352 (0.270, 0.433)	<0.001
Gender	-0.018 (-0.199, 0.163)	0.846
Age	0.007 (-0.001, 0.015)	0.091
BMI	-0.022 (-0.042, -0.002)	0.033
Smoking status	0.041 (-0.108, 0.191)	0.586
Drinking status	-0.232 (-0.699, 0.235)	0.332
**White blood cells**		
WQS index	0.018 (-0.017, 0.053)	0.307
Gender	0.012 (-0.065, 0.089)	0.758
Age	-0.002 (-0.005,0.002)	0.331
BMI	0.015 (0.006, 0.023)	0.001
Smoking status	0.102 (0.038, 0.166)	0.002
Drinking status	0.003 (-0.197, 0.203)	0.977
**TNF-α/WBC**		
WQS index	0.287 (0.199, 0.373)	<0.001
Gender	0.007 (-0.188, 0.203)	0.941
Age	0.008 (-0.001, 0.016)	0.072
BMI	-0.036 (-0.058, -0.015)	0.001
Smoking status	-0.073 (-0.236, 0.088)	0.375
Drinking status	-0.208 (-0.715, 0.298)	0.421
**Serum creatinine**		
WQS index	0.124 (0.098, 0.150)	<0.001
Gender	0.256 (0.194, 0.150)	<0.001
Age	-0.001 (-0.004, 0.001)	0.296
BMI	0.001 (-0.005, 0.008)	0.680
Smoking status	0.040 (-0.012, 0.091)	0.132
Drinking status	0.010 (-0.060, 0.259)	0.222
**eGFR**		
WQS index	-0.115 (-0.138, -0.092)	<0.001
Gender	0.014 (-0.041, 0.068)	0.622
Age	-0.006 (-0.009, -0.004)	<0.001
BMI	0.001 (-0.005, 0.007)	0.792
Smoking status	-0.041 (-0.086, 0.004)	0.078
Drinking status	-0.099 (-0.241, 0.042)	0.170
